# Joint Design of Hybrid Beamforming and Phase Shifts for IRS-Assisted Multi-User mmWave Systems

**DOI:** 10.3390/s26010274

**Published:** 2026-01-01

**Authors:** Ran Zhang, Ye Wang

**Affiliations:** 1 School of Physics and Electronic Information, Anhui Normal University, Wuhu 241002, China; zhrpeace@ahnu.edu.cn; 2 State Key Laboratory of Millimeter Waves, Southeast University, Nanjing 210096, China; 3 Anhui Engineering Research Center on Information Fusion and Control of Intelligent Robot, Wuhu 241002, China

**Keywords:** intelligent reflecting surface, mmWave, beamforming, multi-user, cross-entropy optimization (CEO)

## Abstract

This paper presents a joint design approach for intelligent reflecting surface (IRS)-assisted multi-user millimeter-wave (mmWave) systems. Our goal is to maximize the sum-rate of all users by optimizing the hybrid beamforming at the base station and the low-resolution phase shifters (e.g., 1 bit) at the IRS. To address this, we first adopt a zero-force (ZF) technique to design fully-digital (FD) beamforming and develop a cross-entropy optimization (CEO) framework-based iterative algorithm to calculate IRS phase shifts. Specifically, in this framework, the probability distributions of IRS elements are updated by minimizing the CE, which can generate a solution close to the optimal one with a sufficiently high probability. Then, based on the obtained FD beamforming, an alternating minimization method is applied to acquire hybrid beamforming. Simulation results show that our proposed joint design scheme can achieve enhanced performance compared to the existing schemes while maintaining a lower computational complexity.

## 1. Introduction

Future communication systems are expected to support various emerging applications and services, such as human-computer interaction, autonomous driving, the internet of everything, artificial intelligence, and virtual reality [1]. These advanced services and applications necessitate high-rate data transmission. As a promising technology, millimeter wave (mmWave) communication with wider available bandwidth has the potential to offer gigabits-per-second communication data rates [2,3,4]. However, owing to the high carrier frequency, the mmWave signal experiences severe path loss as compared to the signal with lower frequency bands. On the other hand, the short wavelength and high directivity make mmWave signals vulnerable to blockage events, resulting in link interruption.

To overcome this challenge, intelligent reflecting surface (IRS), which is a reconfigurable planar array composed of massive passive reflecting elements, has been introduced into mmWave communication systems [5,6,7]. More precisely, by independently controlling the coefficient of each IRS element, the incident signal from the base station (BS) is reflected to the desired direction, thereby improving the quality of the received signals and coverage of mmWave communication. In addition, the IRS is also low-cost and energy-efficient without requiring any extra radio frequency (RF) chain.

Recently, IRS-assisted mmWave communications have drawn significant research attention. Specifically, the IRS-assisted single-user mmWave system was investigated in [8,9,10,11,12]. The authors of [8] adopted the semidefinite relaxation (SDR) technique to optimize the problem of reflecting beamforming design. Then, ref. [9] introduced a branch-and-bound (BnB) algorithm to obtain globally optimal solutions for both active and passive beamforming at the access point (AP) and the IRS. In [10], a geometric mean decomposition-based beamforming algorithm was presented to enhance bit error rate (BER) performance in IRS-aided mmWave hybrid MIMO systems. Leveraging the asymptotic orthogonality of array response vectors, the authors in [11] proposed a joint design of the IRS reflection matrix and hybrid beamforming. Ref. [12] reframed the IRS design as a more tractable optimization problem and developed a Riemannian manifold optimization (RMO)-based scheme. Further extending to multi-user scenarios, works such as refs. [13,14,15,16,17,18,19] explored the general IRS-aided multi-user communication systems. Ref. [13] introduced a gradient-projection method aimed at minimizing the mean square error (MSE) between the received symbols and the transmitted symbols. The weighted sum rate maximization problem was studied in [14] via a block coordinate descent algorithm to obtain high-quality suboptimal solution. With a focus on double IRS configurations, ref. [15] proposed an alternating optimization strategy for designing transmit beamforming at the BS and phase shifts at the IRSs. Notably, the aforementioned studies primarily assume continuous phase shifters (PSs), which are often impractical due to hardware cost and power constraints. In response, ref. [16] proposed a successive refinement (SR) algorithm that iteratively optimizes discrete phase shifts element by element. Then, ref. [17] developed a low-convexity machine learning-based beamforming design, and ref. [18] proposed a two-layer penalty-based algorithm to decouple optimization variables in SINR constraints, incorporating manifold optimization for discrete phase shift design. To further enhance system performance under the SR algorithm, ref. [19] presented a coordinate descent method (CDM)-based approach for reflecting beamforming design. Nevertheless, this performance improvement comes at the cost of sacrificing the computational complexity.

In this paper, we focus on an IRS-assisted multi-user mmWave communication system. Our goal is to maximize the overall sum-rate for all users through the joint optimization of hybrid beamforming at the BS and low-resolution phase shifters (e.g., 1 bit) at the IRS. To tackle this challenging non-convex optimization problem, we first decouple the design of the IRS from the hybrid beamforming using the zero-force (ZF) technique. Then, we adopt the idea of the cross-entropy optimization (CEO) framework, which was developed originally for the machine learning domain, to address the IRS design subproblem. Specifically, this framework starts with random sampling based on the probability distributions of IRS elements. After that, it successively refines these probability distributions by minimizing the cross-entropy, progressively yielding a solution that converges toward the optimum with a sufficiently high probability. In addition, according to the obtained ZF beamforming, an alternating minimization method is applied to acquire hybrid beamforming. Simulation results demonstrate that our proposed scheme can achieve better performance than the existing schemes, while at a much lower computational complexity.

Notation: Throughout this paper, matrices are denoted by bold uppercase letters, vectors by bold lowercase letters, and scalars by regular italic letters. The symbols ${\left(\cdot \right)}^{T}$, ${\left(\cdot \right)}^{H}$, and ${\left(\cdot \right)}^{-1}$ represent the transpose, Hermitian transpose, and matrix inverse, respectively. The Frobenius norm is expressed as ${∥\cdot ∥}_{F}$, and $I_{n}$ denotes the n×n identity matrix. The entry in the *i*-th row and *j*-th column of matrix A is written as ${\left[A\right]}_{i,j}$. Statistical expectation is denoted by E[·], and $C^{x\times y}$ represents the space of x×y complex-valued matrices.

## 2. System Model and Problem Formulation

### 2.1. System Model

**Figure 1:**
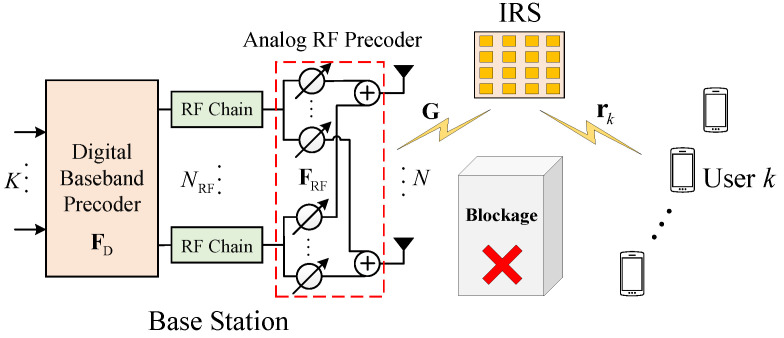
IRS-assisted multi-user mmWave system with hybrid beamforming.

We focus on an IRS-assisted multi-user mmWave system, as illustrated in Figure 1, where a BS equipped with *N* antennas and $N_{\mathrm{RF}}$ RF chains simultaneously serves with *K* single-antenna users through an IRS with *M* reflecting elements. In contrast to the fully-digital (FD) beamforming architecture, the BS employs a hybrid beamforming structure to lower hardware cost and power consumption. Assuming that the direct path between the BS and the users is blocked, the received signal at all *K* users, after the reflection from IRS, is given by the following:

(1) $$\begin{array}{c} y=R\Phi GF_{\mathrm{RF}}F_{D}s+n, \end{array}$$

where $s\in C^{K\times 1}$ denotes the transmitted symbol vector satisfying $E[ss^{H}]=I$. The matrices $F_{D}=\left[d_{1},\ldots ,d_{K}\right]\in C^{N_{\mathrm{RF}}\times K}$ and $F_{\mathrm{RF}}\in C^{N\times N_{\mathrm{RF}}}$ are the digital baseband precoder and analog RF precoder, respectively. The vector $n\in C^{K\times 1}∼\mathrm{CN}\left(0,\sigma_{k}^{2}I\right)$ represents the additive Gaussian noise. The channel matrix from the BS to the IRS is denoted by $G\in C^{M\times N}$, and $R={\left[r_{1},\ldots ,r_{K}\right]}^{H}\in C^{K\times M}$ is the channel matrix from the IRS to the users, with $r_{k}\in C^{M\times 1}$ being the channel vector to the *k*-th user. The phase shift matrix of the IRS is defined as $\Phi =\mathrm{diag}\left(v\right)\in C^{M\times M}$, where $v={\left\{v_{m}\right\}}_{m=1}^{M}={\left[e^{j\theta_{1}},e^{j\theta_{2}},\ldots ,e^{j\theta_{M}}\right]}^{T}$ and $\theta_{m}\in \left(0,2\pi \right)$ is the phase shift introduced by the *m*-th IRS reflecting element.

The signal-to-interference-plus-noise ratio (SINR) at the k-th user can be expressed as follows:

(2) $$\begin{array}{c} \gamma_{k}=\frac{|r_{k}^{H}\Phi GF_{\mathrm{RF}}d_{k}{|}^{2}}{\sum_{i=1,i\neq k}^{K}{\left|r_{k}^{H}\Phi GF_{\mathrm{RF}}d_{i}\right|}^{2}+\sigma_{k}^{2}}. \end{array}$$

Note that the hybrid beamforming requires satisfying the normalized power constraint $\left|\right|F_{\mathrm{RF}}F_{D}{\left|\right|}_{F}^{2}\leq \rho$, where ρ is the transmit power. Considering the practical hardware implementation limit, analog precoding and IRS reflecting elements should be realized by using the discrete PSs. Each element of the *Q*-bit resolution discrete PSs is restricted to the values of a set $F_{Q}=\left\{e^{j0},e^{j△\theta },\ldots ,e^{j(2^{Q}-1)△\theta }\right\}$, where $△\theta =\frac{2\pi }{2^{Q}}$. Thus, we can obtain the constraints $v_{m}\in F_{Q_{1}}$ and ${\left[F_{\mathrm{RF}}\right]}_{i,j}\in F_{Q_{2}}$, ∀i,j, where $Q_{1}$ and $Q_{2}$ denote the resolution of IRS and analog precoding, respectively.

### 2.2. Channel Model

To capture the limited scattering features of mmWave propagation, we adopt the Saleh–Valenzuela channel model. Assuming the channel state information (CSI) of all links can be obtained by existing channel estimation schemes [20], the channel model with $L_{1}$ propagation paths for the BS-IRS links can be written as follows:

(3) $$\begin{array}{c} G=\sqrt{\frac{NM}{L_{1}}}\sum_{l=1}^{L_{1}}\alpha_{l}a_{\mathrm{IRS}}\left(\omega_{l}^{r},\phi_{l}^{r}\right)a_{\mathrm{BS}}^{H}\left(\eta_{l}^{t}\right), \end{array}$$

where $\alpha_{l}$ denotes the complex, $\omega_{l}^{r},\phi_{l}^{r}$ and $\eta_{l}^{t}$ are azimuthal angle of arrival (AoA), elevational AoA, and angle of departure (AoD) of the *l*-th path between the BS and the IRS, respectively. The functions $a_{\mathrm{BS}}\left(\eta \right)$ and $a_{\mathrm{IRS}}\left(\omega ,\phi \right)$ are respectively the array steering vectors at the BS and the IRS, which can be given by the following:

(4) $$\begin{array}{c} a_{\mathrm{BS}}\left(\eta \right)=\frac{1}{\sqrt{N}}{\left[1,\ldots ,e^{j\frac{2\pi d}{\lambda }n\sin\left(\eta \right)},\ldots ,\right]}^{T}, \end{array}$$

(5) $$\begin{array}{c} a_{\mathrm{IRS}}\left(\omega ,\phi \right)=\frac{1}{\sqrt{M}}{\left[1,\ldots ,e^{j\frac{2\pi d}{\lambda }\left(x\sin\left(\omega \right)\sin\left(\phi \right)+y\cos\left(\phi \right)\right)},\ldots ,\right]}^{T}, \end{array}$$

where 0≤n≤(N−1), $0\leq \left\{x,y\right\}\leq \left(\sqrt{M}-1\right)$. λ and *d* are the signal wavelength and the antenna spacing. The channel vector $r_{k}\in C^{M\times 1}$ between the IRS and the *k*-th user, can be written as follows

(6) $$\begin{array}{c} r_{k}=\sqrt{\frac{M}{L_{2}}}\sum_{l=1}^{L_{2}}\beta_{k,l}a_{\mathrm{IRS}}\left(\omega_{l}^{t},\phi_{l}^{t}\right), \end{array}$$

where $L_{2}$ is the propagation paths for the IRS-users links. $\beta_{k,l}$, $\omega_{l}^{t}$ and $\phi_{l}^{t}$ are the complex, azimuthal AoD, and elevational AoD of the *l*-th path for the *k*-th user, respectively.

### 2.3. Problem Formulation

In this paper, our objective is to maximize the sum-rate of all the *K* users by jointly designing the digital baseband precoding matrix $F_{D}$, the analog precoding matrix $F_{\mathrm{RF}}$, and the IRS phase shift vector v. The optimization problem can be formulated as follows:

(7) $$\begin{array}{c} P_{1}:\;\underset{\left\{F_{D},F_{\mathrm{RF}},v\right\}}{\mathrm{maximize}}\;f\left(F_{D},F_{\mathrm{RF}},v\right)=\sum_{k=1}^{K}\log_{2}\left(1+\gamma_{k}\right)\\ s.t.\left\{\begin{array}{c} ∥F_{\mathrm{RF}}{F_{D}∥}_{F}^{2}\leq \rho ,\\ {\left[F_{\mathrm{RF}}\right]}_{i,j}\in F_{Q_{2}},\forall i,j,\\ v_{m}\in F_{Q_{1}},\forall m. \end{array}\right. \end{array}$$

Such an optimization problem is intractable, owing to the correlation among $F_{D}$, $F_{\mathrm{RF}}$, and v. To simplify this problem, we first consider an IRS-assisted multi-user mmWave system with an FD beamforming architecture, namely, $F=F_{\mathrm{RF}}F_{D}=\left[f_{1},\ldots ,f_{K}\right]$. When the optimal F is obtained, we can adopt an alternating minimization method in [21] to search for a hybrid beamforming matrix ($F_{\mathrm{RF}}F_{D}$) to approach the optimal F. Thus, the corresponding problem can be rewritten as follows:

(8) $$\begin{array}{c} P_{2}:\;\underset{\left\{F,v\right\}}{\mathrm{maximize}}\;f\left(F,v\right)=\sum_{k=1}^{K}\log_{2}\left(1+\gamma_{k}\right)\\ s.t.\left\{\begin{array}{c} {∥F∥}_{F}^{2}\leq \rho ,\\ v_{m}\in F_{Q_{1}},\forall m. \end{array}\right. \end{array}$$

However, problem $P_{2}$ is still challenging due to the coupled F and v. Therefore, we will develop a ZF-based CEO design algorithm to solve problem $P_{2}$. The details are presented in the next section.

## 3. Algorithm Design

In this section, we propose a ZF-based CEO algorithm to design IRS phase shift and FD beamforming by an alternating iterative procedure. After that, we utilize an alternating minimization method to optimize the hybrid beamforming matrix so that it could approach the performance of the FD beamforming matrix.

### 3.1. ZF-Based CEO Algorithm Design

The CEO framework is a probabilistic model-based iterative optimization algorithm. A key advantage of this framework is to optimize large-scale discrete variable spaces, which are inherent to IRS design problems. In addition, it can achieve substantially lower computational complexity relative to conventional exhaustive search and branch-and-bound techniques. This framework consists of the following key steps: (i) **Sampling:** Generating several random samples of candidate solutions based on a specified probability distribution. (ii) **Selection:** By sorting the objective function values in descending order, some top-performing candidate solutions are selected as the elite samples. (iii) **Updating:** Based on the selected elites, the probability distribution parameters are updated by minimizing the CE. (iv) **Iteration:** Repeat sampling and updating until convergence.

To characterize the sampling behavior in the CEO framework, we first define a probability parameter set ${\left\{p_{m,q}\right\}}_{q=1}^{2^{Q_{1}}}$, where $p_{m,q}=\mathrm{Pr}\left(v_{m}=e^{jq△\theta }\right)$ denotes the probability that $v_{m}$ selects the *q*-th element from set $F_{Q_{1}}$, and $\sum_{q=1}^{2^{Q_{1}}}p_{m,q}=1$. In the initialization phase, we assume that all the IRS phase shifts belong to $F_{Q_{1}}$ with equal probability. At the *i*-th iteration, we generate *A* candidate IRS phase shifts ${\left\{v^{\left[a\right]}\right\}}_{a=1}^{A}$ according to the probability distribution $H(v^{\left[a\right]};P^{\left(i\right)})$, written as follows:

(9) $$\begin{array}{c} H\left(v^{\left[a\right]};P^{\left(i\right)}\right)=\prod_{m=1}^{M}\sum_{q=1}^{2^{Q_{1}}}p_{m,q}^{\left(i\right)}1_{\left\{v_{m}^{\left[a\right]}={\left[F_{Q_{1}}\right]}_{q}\right\}}, \end{array}$$

where P is a matrix composed of $p_{m,q}$, m∈{1,…,M}, $q\in \{1,\ldots 2^{Q_{1}}\}$. $1_{\left\{\cdot \right\}}$ denotes the indicator function for an event, and ${\left[F_{Q_{1}}\right]}_{q}$ is the *q*-th element of $F_{Q_{1}}$. When any IRS phase shift $v^{\left[a\right]}$ is given, the sum-rate maximization problem $P_{2}$ can be written as follows:

(10) $$\begin{array}{c} P_{3}:\;\underset{F}{\mathrm{maximize}}\;f\left(F\right)=\sum_{k=1}^{K}\log_{2}\left(1+\gamma_{k}\right)\\ {s.t.\;∥F∥}_{F}^{2}\leq \rho , \end{array}$$

By using a ZF technique, the FD beamforming matrix can be computed by the following:

(11) $$\begin{array}{c} F_{\mathrm{ZF}}=H_{e}^{H}{\left(H_{e}H_{e}^{H}\right)}^{-1}, \end{array}$$

where $H_{e}≜R\Phi G$. To meet the transmit power constraint, the optimal FD beamforming matrix is given as follows:

(12) $$\begin{array}{c} F^{★}=\frac{\sqrt{\rho }}{∥F_{\mathrm{ZF}}{∥}_{F}}F_{\mathrm{ZF}} \end{array}$$

According to the obtained FD beamforming matrix $F^{★}$, problem $P_{2}$ can be reformulated as follows:

(13) $$\begin{array}{c} P_{4}:\;\underset{v}{\mathrm{maximize}}\;f\left(v\right)=\sum_{k=1}^{K}\log_{2}\left(1+\gamma_{k}\right)\\ s.t.\;v\in \{v^{\left[1\right]},\ldots ,v^{\left[A\right]}\}, \end{array}$$

Then, we calculate *A* sum-rate ${\left\{f\left(v^{\left[a\right]}\right)\right\}}_{a=1}^{A}$ corresponding to the candidate IRS phase shifts, and sort these values ${\left\{f\left(v^{\left[a\right]}\right)\right\}}_{a=1}^{A}$ in descending order, which is written as follows:

(14) $$\begin{array}{c} f\left(v^{\left[1\right]}\right)\geq f\left(v^{\left[2\right]}\right)\geq \ldots \geq f\left(v^{\left[A_{elite}\right]}\right)\geq \ldots \geq f\left(v^{\left[A\right]}\right) \end{array}$$

Note that only the top-performing candidate solutions are considered in the CEO framework. Therefore, we choose $A_{elite}$ IRS phase shifts with the largest sum-rate as elites to update $p_{m,q}^{\left(i+1\right)}$ by minimizing CE, which is equivalent to the following [22]:

(15) $$\begin{array}{c} P_{5}:\;p_{m,q}^{\left(i+1\right)}=\underset{p_{m,q}^{\left(i\right)}}{\mathrm{argmax}}\;\frac{1}{A}\sum_{a=1}^{A_{\mathrm{elite}}}\ln H\left(v^{\left[a\right]};P^{\left(i\right)}\right)\\ s.t.\;\sum_{q=1}^{2^{Q_{1}}}p_{m,q}^{\left(i\right)}=1,\forall m. \end{array}$$

After that, we adopt the Lagrange multiplier method to solve problem $P_{5}$. Specifically, the Lagrange function associated with (15) can be expressed as follows:

(16) $$\begin{array}{c} L=\frac{1}{A}\sum_{a=1}^{A_{\mathrm{elite}}}\ln H\left(v^{\left[a\right]};P^{\left(i\right)}\right)+\sum_{m=1}^{M}\mu_{m}\left(\sum_{q=1}^{2^{Q_{1}}}p_{m,q}^{\left(i\right)}-1\right), \end{array}$$

where $\mu_{m}$ is the Lagrange multiplier. The partial derivatives of the Lagrange function on $p_{m,q}^{\left(i\right)}$ is derived as follows:

(17) $$\begin{array}{c} \frac{\partial L}{\partial p_{m,q}^{\left(i\right)}}=\frac{1}{A}\sum_{a=1}^{A_{\mathrm{elite}}}1_{\left\{v_{m}^{\left[a\right]}={\left[F_{Q_{1}}\right]}_{q}\right\}}+\mu_{m}p_{m,q}^{\left(i\right)}, \end{array}$$

Setting $\frac{\partial L}{\partial p_{m,q}^{\left(i\right)}}=0$, we can obtain the Lagrange multiplier as follows:

(18) $$\begin{array}{c} \mu_{m}=-\frac{A_{elite}}{A}, \end{array}$$

The probability $p_{m,q}^{\left(i+1\right)}$ can be updated by the following:

(19) $$\begin{array}{c} p_{m,q}^{\left(i+1\right)}=\frac{\sum_{a=1}^{A_{\mathrm{elite}}}1_{\left\{v_{m}^{\left[a\right]}={\left[F_{Q_{1}}\right]}_{q}\right\}}}{A_{elite}}, \end{array}$$

Finally, a smoothing process is adopted to avoid the local optimum, which is given by the following:

(20) $$\begin{array}{c} P^{\left(i+1\right)}=\varpi^{\left(i+1\right)}\times P^{\left(i+1\right)}+\left(1-\varpi^{\left(i+1\right)}\right)\times P^{\left(i\right)}. \end{array}$$

where 0<ϖ<1 is the smoothing step size. Repeating the above procedure, the probability distribution H(v;P) will be refined to generate a solution close to the optimal one with a sufficiently high probability. The proposed ZF-based CEO algorithm design is summarized in Algorithm 1, where *I* is the maximum number of iterations.
**Algorithm 1** Proposed ZF-based CEO algorithm for solving (8).1:**Input:** $R,G,A,A_{\mathrm{elite}}$, and *I*.2:**Initialization:** $i=0,\;p_{m,q}^{\left(0\right)}=\frac{1}{2^{Q_{1}}}$.3:**for** i = 0 **to** *I* **do**4:    Generate *A* candidate v randomly as ${\left\{v^{\left[a\right]}\right\}}_{a=1}^{A}$ based on the $H(v^{\left[a\right]};P^{\left(i\right)})$;5:    Compute *A* corresponding FD beamforming matrices ${\left\{F^{\left[a\right]}\right\}}_{a=1}^{A}$ based on the effective channel $H_{e}^{\left[a\right]}≜R\mathrm{diag}\left(v^{\left[a\right]}\right)G$ as in (12);6:    Calculate the sum-rate ${\left\{f\left(v^{\left[a\right]}\right)\right\}}_{a=1}^{A}$ using (8) and sort them in descending as $$f\left(v^{\left[1\right]}\right)\geq f\left(v^{\left[2\right]}\right)\geq \ldots \geq f\left(v^{\left[A_{\mathrm{elite}}\right]}\right)\geq \ldots \geq f\left(v^{\left[A\right]}\right);$$7:   Select elites as $v^{\left[1\right]},v^{\left[2\right]},\ldots ,v^{\left[A_{\mathrm{elite}}\right]}$;8:   Update $P^{\left(i+1\right)}$ according to (20);9:    i=i+1;10:**end**11:**Output:** IRS phase shift vector $v^{\left[1\right]}$, FD beamforming matrix $F^{★}$.

**Remark** **1.** *It has been verified in [23,24,25,26] that through appropriate selection of the smoothing parameter in (20), the CEO-based framework, utilizing discrete sampling distributions, can achieve probabilistic convergence to the global optimum, guaranteeing a probability of 1 for obtaining the optimal IRS phase shift. Ref. [26] shows more details of the theoretical convergence analysis of the CEO framework.*

### 3.2. Hybrid Beamforming Design

Based on the obtained IRS phase shift vector and the optimal FD beamforming matrix, the hybrid beamforming design problem can be formulated as follows:

(21) $$\begin{array}{c} P_{6}:\;\underset{\left\{F_{D},F_{\mathrm{RF}}\right\}}{\mathrm{minimize}}\;||F^{★}-F_{\mathrm{RF}}F_{D}{\left|\right|}_{F}^{2}\\ s.t.\left\{\begin{array}{c} ∥F_{\mathrm{RF}}F_{D}{∥}_{F}^{2}\leq \rho ,\\ {\left[F_{\mathrm{RF}}\right]}_{i,j}\in F_{Q_{2}},\forall i,j, \end{array}\right. \end{array}$$

To simplify this problem, the hybrid beamforming matrix is rewritten as follows:

(22) $$\begin{array}{c} F_{\mathrm{RF}}F_{D}={\left[F_{\mathrm{RF}}\right]}_{:,1}{\left[F_{D}\right]}_{1,:}+\ldots +{\left[F_{\mathrm{RF}}\right]}_{:,n}{\left[F_{D}\right]}_{n,:}+\ldots +{\left[F_{\mathrm{RF}}\right]}_{:,N_{\mathrm{RF}}}{\left[F_{D}\right]}_{N_{\mathrm{RF}},:} \end{array}$$

where $n\in \{1,2,\ldots ,N_{\mathrm{RF}}\}$. Assuming that both $F_{\mathrm{RF}}$ and $F_{D}$ are given except the column vector ${\left[F_{\mathrm{RF}}\right]}_{:,n}$ and the row vector ${\left[F_{D}\right]}_{n,:}$, the hybrid beamforming problem can be decomposed into $N_{\mathrm{RF}}$ sub-problems. The *n*-th subproblem is expressed as follows:

(23) $$\begin{array}{c} P_{7}:\;\underset{\left\{{\left[F_{D}\right]}_{n,:},{\left[F_{\mathrm{RF}}\right]}_{:,n}\right\}}{\mathrm{minimize}}\;||F_{n}-F_{\mathrm{RF}}F_{D}{\left|\right|}_{F}^{2}\\ s.t.\left\{\begin{array}{c} ∥F_{\mathrm{RF}}F_{D}{∥}_{F}^{2}\leq \rho ,\\ {\left[F_{\mathrm{RF}}\right]}_{i,j}\in F_{Q_{2}},\forall i,j, \end{array}\right. \end{array}$$

where $F_{n}=F^{★}-F_{\mathrm{RF}}F_{D}+{\left[F_{\mathrm{RF}}\right]}_{:,n}{\left[F_{D}\right]}_{n,:}$ is defined as an auxiliary matrix. Specifically, we adopt an alternating minimization method of [21] to optimize ${\left[F_{\mathrm{RF}}\right]}_{:,n}$ and ${\left[F_{D}\right]}_{n,:}$, and then update $F_{n}$. This process continues until all column vectors of $F_{\mathrm{RF}}$ and all row vectors of $F_{D}$ are obtained. Notably, the alternating minimization procedure is well-behaved and robust, with its convergence to a critical point having been proven in prior work [21].

### 3.3. Comparison of Computational Complexity

In this subsection, we analyze the computational complexity of the proposed ZF-CEO algorithm and choose the CDM [19] and SR [16] algorithms for comparison. For the first stage of the proposed ZF-CEO algorithm, the dominant cost lies in computing the effective channel matrix (Step 5), which requires O(KA(MN+M)) operations. In the second stage, applying the zero-forcing technique to obtain the optimal fully-digital beamforming matrix dominates the complexity, with a cost of $O(A\left(K^{2}N+K^{3}\right))$. The overall complexity of the ZF-CEO algorithm is $O(A\left(KMN+KM+K^{2}N+K^{3}\right))$. For the SR algorithm, the total complexity is $O(2^{Q_{1}}\left(KMN+K^{2}M+K^{3}\right))$. The total complexity of the CDM algorithm is $O(2^{Q_{1}}\left(MN^{3}+2KMN^{2}+MN+KMN\right))$. Assuming {M,N}≫K, the dominant computational complexity of each algorithm is summarized in Table 1. It can be observed that the complexity of the proposed ZF-CEO algorithm is of the same order as that of the SR algorithm, while being slightly lower than that of the CDM algorithm. This indicates that the computational complexity of our proposed algorithm is practically an acceptable cost for IRS-assisted multi-user mmWave communication systems. In addition, we present the runtime of the proposed ZF-CEO, SR, and CDM algorithms in Table 1. It can be seen that the runtime of our algorithm is comparable to that of the other two algorithms. This is because the iterative sampling and probability updates involved in the CEO framework introduce non-negligible constant overhead, despite the relatively lower theoretical complexity of our proposed algorithm.

## 4. Simulation Results

We conduct the simulation results to evaluate the performance of the proposed algorithm in this section. For simulation, a three-dimensional geometry is adopted: a uniform linear array (ULA) at the BS and a uniform planer array (UPA) at the IRS are located in the x−z plane and the y−z plane, respectively. *K* single-antenna users are randomly distributed within a circular area centered at (0, 148 m). Considering the channel modeling realism and the imperfect CSI, the random angular offset parameters $\epsilon_{1}\in \left[-3^{\circ },3^{\circ }\right]$ and $\epsilon_{2}\in \left[-5^{\circ },5^{\circ }\right]$ are, respectively, introduced to the AoA and AoD. Moreover, consistent with the parameter configuration established in [19], the other simulation parameter values are summarized in Table 2.

**Figure 2:**
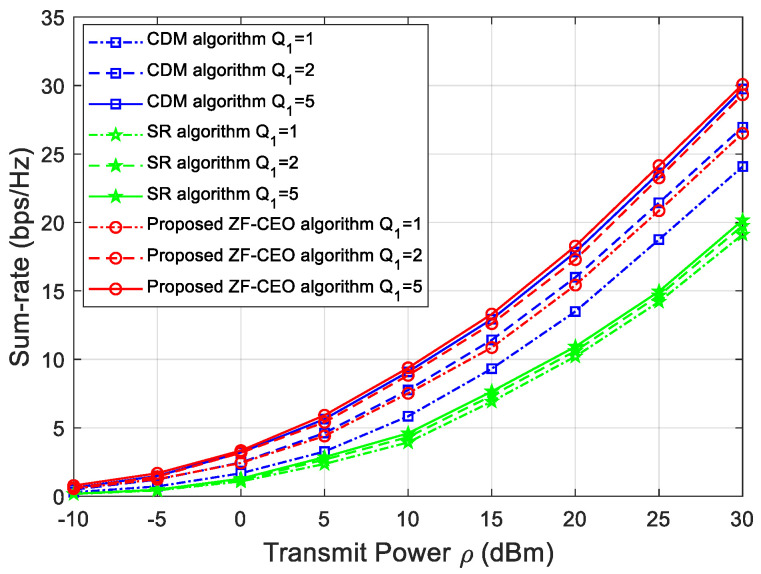
Sum-rate versus the transmit power for $Q_{1}=1$ bit, $Q_{1}=2$ bit, and $Q_{1}=5$ bit.

Figure 2 shows the sum-rate versus the transmit power for $Q_{1}=1$ bit, $Q_{1}=2$ bit, and $Q_{1}=5$ bit phase shift resolutions. The proposed ZF-CEO algorithm is evaluated against two benchmark schemes: (1) the CDM algorithm [19]; (2) the SR algorithm [16], which has been widely used as a performance baseline for IRS-assisted multi-user systems. As observed in Figure 2, the sum-rate rises with increasing transmit power across all algorithms. Furthermore, when the resolution of IRS $Q_{1}$ increases, the sum-rate performance will increase over the entire considered ρ range. Most importantly, the proposed ZF-CEO algorithm outperforms the CDM and the SR algorithms when $Q_{1}=5$ bits, and the performance gap becomes more pronounced when $Q_{1}=1$ bit. For instance, at a transmit power of 20 dBm and $Q_{1}=1$ bit, the gain of ZF-CEO exceeds 30% and 10% compared to SR and CDM algorithms. In conclusion, the proposed ZF-CEO algorithm can achieve a reasonable trade-off. It offers a significant performance advantage in low-resolution scenarios (e.g., 1 bit) with only a marginal increase in practical runtime compared to the existing algorithms.

**Figure 3:**
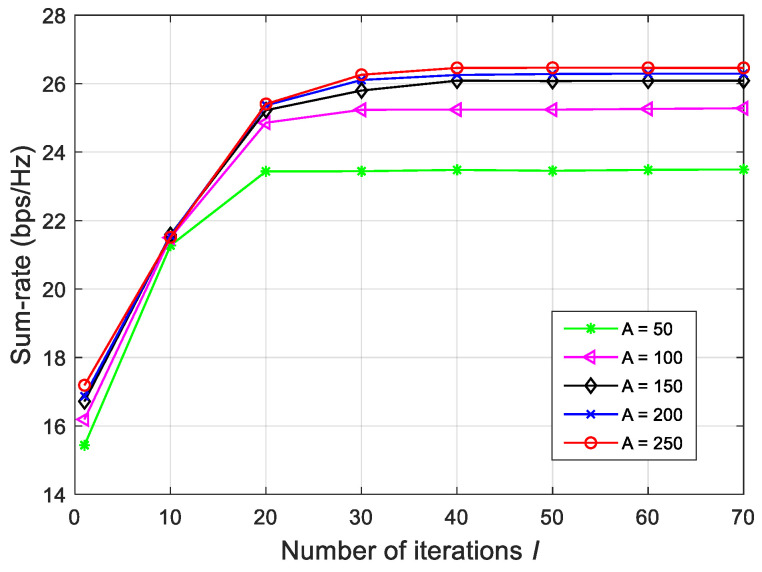
Sum-rate against the number of candidates and the number of iterations for $Q_{1}=1$ bit and ρ=30 dBm.

Next, we examine the convergence behavior of our proposed ZF-CEO algorithm, as illustrated in Figure 3. Figure 3 plots the sum-rate against the number of iterations *I* and the number of candidates *A*, with parameters set as $A_{elite}/A=0.2$, $Q_{1}=1$ bit, and ρ=30 dBm. It can be seen that the sum-rate first rapidly increases and then stabilizes after a modest number of iterations (e.g., I=40), confirming the efficiency of the proposed ZF-CEO algorithm. Moreover, while increasing *A* yields noticeable sum-rate gains when *A* is relatively small, this improvement diminishes once *A* exceeds a certain threshold. This suggests that a moderately large candidate size (e.g., A=150) is sufficient to achieve near-optimal performance.

**Figure 4:**
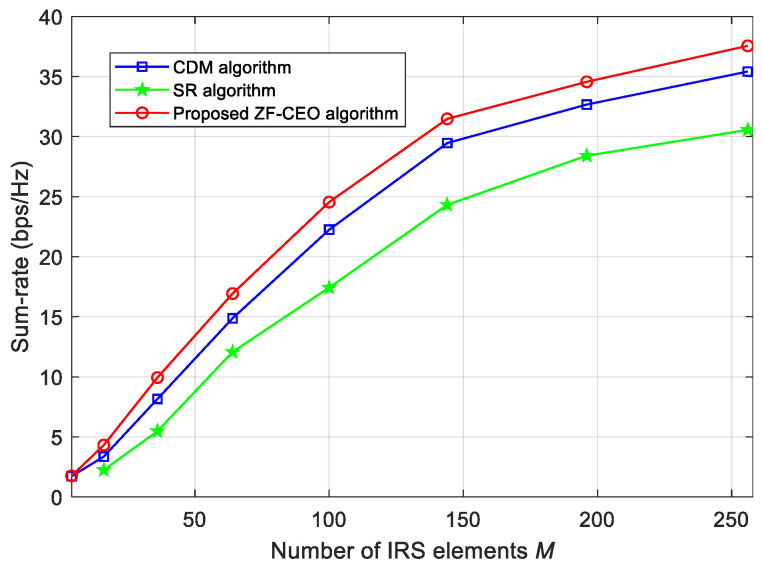
Sum-rate of different algorithms against the number of IRS elements for $Q_{1}=1$ bit and ρ=30 dBm.

Figure 4 illustrates the sum-rate of different algorithms against the number of IRS elements M={16,36,64,100,144,196,256}, under $Q_{1}=1$ bit and ρ=30 dBm. As expected, the sum-rate performance of all algorithms grows as the number of IRS elements *M*. However, the performance gain diminishes as the number of IRS elements increases. In Figure 4, we can see that the proposed ZF-CEO algorithm achieves enhanced performance compared to the CDM and SR algorithms across the considered range of *M*, with the performance gap widening slightly as *M* grows larger. This trend confirms that the proposed ZF-CEO method offers greater benefits in systems equipped with a larger IRS.

**Figure 5:**
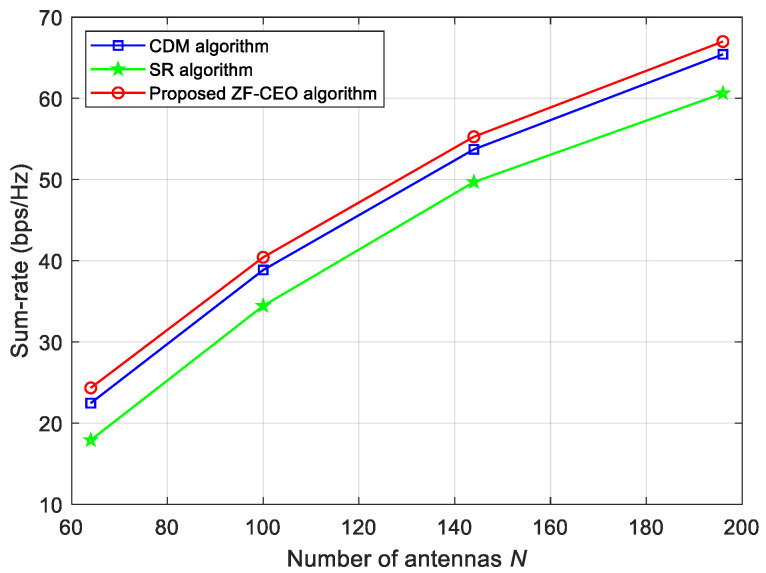
Sum-rate versus the number of antennas at the BS for $Q_{1}=1$ bit and ρ=30 dBm.

In Figure 5, we plot the achievable sum-rate for N={64,100,144,196} when $Q_{1}=1$ bit and ρ=30 dBm. It is observed that the sum-rate of all algorithms improves with increasing *N*. This indicates that the sum-rate performance of the proposed ZF-CEO algorithm can be improved by deploying more antennas at the BS. As shown in Figure 5, the proposed ZF-CEO algorithm has better performance than the CDM and SR algorithms over the whole *N* range in consideration. For example, at N=100, the performance gains of the proposed ZF-CEO algorithm over the CDM and SR algorithms are approximately 4.47% and 17.6%, respectively.

## 5. Conclusions

In this paper, we studied an IRS-assisted multi-user mmWave system, aiming to maximize the overall sum-rate through joint optimization of the BS hybrid beamforming and the low-resolution phase shifters (e.g., 1 bit) at the IRS. To tackle this non-convex problem, the IRS design was first decoupled from the hybrid beamforming process using a ZF technique. Then, we developed a CEO framework-based iterative algorithm to calculate the discrete phase shifts of IRS. Simulation results showed that our proposed joint design scheme can improve the sum-rate performance, particularly under low-resolution phase shifter constraints, while at a much lower computational complexity.

## Figures and Tables

**Table 1:** Comparison of computational complexity.

Algorithm	Dominant Complexity (per Iteration)	Runtime (s)
ZF-CEO	$O(A\left(KMN+KM+K^{2}N+K^{3}\right))$	1.26
SR	$O(2^{Q_{1}}\left(KMN+K^{2}M+K^{3}\right))$	1.32
CDM	$O(2^{Q_{1}}\left(MN^{3}+2KMN^{2}+MN+KMN\right))$	1.38

**Table 2:** Simulation parameters.

Parameters	Assumption
BS location ($x_{\mathrm{BS}}$, $y_{\mathrm{BS}}$, $z_{\mathrm{BS}}$)	(2 m, 0, 10 m)
IRS location ($x_{\mathrm{IRS}}$, $y_{\mathrm{IRS}}$, $z_{\mathrm{IRS}}$)	(0, 148 m, 10 m)
Number of users	K=4
Number of antennas	N=64
Number of reflecting elements	M=100
Number of propagation paths	$L_{1}=L_{2}=7$
Azimuth AoA/AoD	$\omega \in \left(-\frac{\pi }{2},\frac{\pi }{2}\right),\eta \in \left(-\frac{\pi }{2},\frac{\pi }{2}\right)$
Elevation AoA	$\phi \in (-\frac{\pi }{2},\frac{\pi }{2})$
Transmit power	ρ=30 dBm
Noise power	$\sigma^{2}=-90$ dBm

## Data Availability

The data presented in this study are available on request from the corresponding author.
